# Targeting Autophagy in Thyroid Cancer: EMT, Apoptosis, and Cancer Stem Cells

**DOI:** 10.3389/fcell.2022.821855

**Published:** 2022-06-29

**Authors:** Tammy M. Holm, Syn Yeo, Kevin M. Turner, Jun-Lin Guan

**Affiliations:** ^1^ Department of Surgery, The University of Cincinnati, Cincinnati, OH, United States; ^2^ Vontz Center for Molecular Studies, Department of Cancer Biology, The University of Cincinnati, Cincinnati, OH, United States

**Keywords:** autophagy, thyroid cancer, EMT, apoptosis, cancer stem cells

## Abstract

Autophagy is a highly conserved recycling process through which cellular homeostasis is achieved and maintained. With respect to cancer biology, autophagy acts as a double-edged sword supporting tumor cells during times of metabolic and therapeutic stress, while also inhibiting tumor development by promoting genomic stability. Accumulating evidence suggests that autophagy plays a role in thyroid cancer, acting to promote tumor cell viability and metastatic disease through maintenance of cancer stem cells (CSCs), supporting epithelial-to-mesenchymal transition (EMT), and preventing tumor cell death. Intriguingly, well-differentiated thyroid cancer is more prevalent in women as compared to men, though the underlying molecular biology driving this disparity has not yet been elucidated. Several studies have demonstrated that autophagy inhibitors may augment the anti-cancer effects of known thyroid cancer therapies. Autophagy modulation has become an attractive target for improving outcomes in thyroid cancer. This review aims to provide a comprehensive picture of the current knowledge regarding the role of autophagy in thyroid cancer, focusing on the potential mechanism(s) through which inhibition of autophagy may enhance cancer therapy and outcomes.

## Introduction

Thyroid cancer is the most common endocrine malignancy. Worldwide, there were 586,202 new cases of thyroid cancer in 2020 (Sung et al., 2021). Well-differentiated thyroid cancers (WDTC) account for the majority of cases and generally exhibit a favorable prognosis with conventional treatment including surgery and possibly radioactive iodine (RAI) ablation. While WDTC is significantly more prevalent in women as compared to men, the underlying molecular biology driving this mutation has not yet been elucidated and recent epidemiologic studies suggest that the observed disparity between men and women is due to overdiagnosis (Song et al., 2013; Meng et al., 2014; Daskalaki et al., 2018; Gugnoni et al., 2021). Importantly, mortality from thyroid cancer increases significantly for patients with recurrent WDTC, which occurs in up to 20% of cases, or those with dedifferentiated, anaplastic thyroid cancer (ATC) (Mazzaferri and Jhiang, 1994). Therapeutic options for advanced, dedifferentiated disease remain limited.

The term “autophagy” from the Greek, self-devouring, was coined by the Belgian biochemist and cytologist, Dr. Christian de Duve. Initially recognized as a protective process induced to prevent starvation, and then a house-keeping pathway for the orderly degradation of unnecessary or dysfunctional cellular components, autophagy is now recognized to play an active role in a multitude of human diseases including cancer (Dikic and Elazar, 2018; Singh et al., 2018). In this review, we examine the current understanding of the role of autophagy in pathogenesis of thyroid cancer with a focus on EMT, apoptosis, and cancer stem cells (CSCs). Moreover, we provide a perspective on how these processes can be targeted for potential therapeutic approaches and summarize the most recent data on the role of autophagy inhibitors in thyroid cancer care (Table 1).

**TABLE 1:** Effects of autophagy modulation in thyroid cancer.

	Thyroid cancer cell lines	Effects of autophagy modulation	References
**Autophagy inhibition**			
CDH-6	• PTC: TPC-1, BCPAP	Autophagy inhibition drives DRP-1 mediated mitochondrial reorganization and induces EMT	Gugnoni et al. (2017)
BIRC7	• PTC: BCPAP, TPC-1, K1, IHH4	Autophagy inhibition via BECN1 and ATG5 repression induces EMT	Liu et al. (2020)
Lys05	• PTC: T32, T68	Autophagy inhibition at the level of the lysosome inhibits EMT in all cell lines and induces apoptosis only in FTC-133 and 8505c cells	Holm et al. (2021)
	• FTC: FTC-133		
	• ATC: 8505c		
BAG3	• PTC: KTC1	Autophagy inhibition in starved cancer cells induces apoptosis	Liu et al. (2013)
	• ATC: FRO, KTC3		
LDHA	• PTC: KTC1, BCPAP	Autophagy inhibition induces EMT and cell proliferation	Hou et al. (2021)
**Autophagy induction**			
BANCR	• PTC: IHH4	Autophagy induction inhibits apoptosis and induces cell proliferation	Wang et al. (2014)
HMGB1	• PTC: TPC-1	Autophagy induction sustains thyroid cell differentiation (NIS expression)	Chai et al. (2019)
	• FTC: FTC-133		
**Dual therapy**			
LDHA inhibition + HCQ	• PTC: BCPAP	LDHA inhibition induces autophagy inhibiting apoptosis. Dual LDHA and autophagy inhibition drives apoptosis, no effect on EMT	Hou et al. (2021)
Vemurafenib + HCQ	• PTC: BCPAP	Autophagy induction with vemurafenib. Synergism with HCQ + vemurafenib increases cell death	Wang et al. (2017)
	• ATC: 8505c		
Apatinib + CQ/HCQ	• PTC: K1, KTC1	Autophagy induction with apatinib. Synergism with CQ/HCQ + apatinib increases apoptosis	Feng et al. (2018) and Meng et al. (2020)
	• ATC: KHM-5M, C643		
Sorafenib + CQ	• FTC: FTC-133	Autophagy and apoptosis induction with sorafenib. Dual therapy with sorafenib + CQ results in synergistic increase in apoptosis	Yi et al. (2018)
	• ATC: 8505c		
Radiation/doxorubicin + RAD001	• PTC: TPC1	Autophagy induced by radiation/doxorubicin. Dual therapy with potent autophagy inducer (RAD001) increased cell death independent of apoptosis	Lin et al. (2009); Lin et al. (2010)
	• ATC: 8505c		

PTC, Papillary thyroid cancer; FTC, Follicular thyroid cancer; ATC, Anaplastic thyroid cancer.

## Autophagy and Epithelial-to-Mesenchymal Transition: Invasion and Stabilization

Epithelial-to-mesenchymal transition (EMT) is considered to be crucial for the development of an invasive phenotype in tumor cells and therefore a hallmark of metastatic disease (Brabletz et al., 2018). Mesenchymal features are transiently adapted by epithelial cells which subsequently lose their apical basal polarity as well as cell-cell adhesion. Further dedifferentiation of epithelial cells results in higher cellular plasticity and motility, as well as resistance to apoptosis. Recently, EMT has been described as a spectrum of states, including intermediary hybrid-EMT states. The process of EMT is highly regulated and requires profound molecular and biochemical changes both within the tumor cell itself and with the surrounding microenvironment.

The SNAI family of transcription factors (TF) including Snail and Slug, together with the zinc-finger E-box-binding homeobox (ZEB) 1/2, and basic helix-loop-helix transcription factors Twist 1/2, are thought to be the drivers of EMT as they promote the expression of genes typically expressed in mesenchymal cells, namely: N-cadherin, vimentin, and fibronectin, while concomitantly suppressing the expression of epithelial markers: E-cadherin, cytokeratins, claudins, and occludins (Meng et al., 2014; Brabletz et al., 2018). The loss of E-cadherin leads to the activation of the Wnt/β-catenin pathway and subsequent transcription of genes promoting proliferation and migration. Consequently, cells undergoing EMT lose polarity and acquire a fibroblast-like morphology which allows for the degradation of the underlying basement membrane and subsequent migration from the epithelial layer and surrounding extracellular matrix. Activation of EMT is thought to be pivotal for both the acquisition of the malignant phenotype and metastases.

The tumor microenvironment is characterized in part by inflammation, with tumor cells recruiting activated fibroblasts and immune cells which in turn secrete tissue-specific soluble factors including transforming growth factor beta (TGF-β) and tissue necrosis factor alpha (TNF-α) (Song et al., 2013). Hypoxia within the microenvironment also promotes EMT via hypoxia-inducible-factor 1-alpha (HIF1α) and increased mitochondrial production of reactive oxygen species (ROS). All of these factors, among several others, have also been shown to influence both the induction and suppression of autophagy (Daskalaki et al., 2018). This observation recently led to multiple studies seeking to describe the intricate relationship between these two processes and cancer. Notably, the effect of autophagy on EMT appears to be dependent on cell-type as well as the specific autophagic stimulus.

Because of the observation that EMT is often induced in cancers with more aggressive phenotypes and heightened metastatic potential, recent interest in the relationship between EMT and autophagy in thyroid cancer is focused on identifying markers that could help predict which thyroid cancers are more likely to progress to recurrent or advanced disease. Several recent studies have utilized large tumor repositories in concert with genomic datasets including The Cancer Genome Atlas (TCGA) to identify potential markers of advanced disease. Multiple studies have then endeavored to understand how these markers interact with autophagy to drive disease progression (Gugnoni et al., 2017; Gugnoni et al., 2021; Hou et al., 2021; Xu et al., 2021).

Cadherin-6 (CDH6) is a type 2, atypical cadherin which is aberrantly reactivated in several cancers (Shimazui et al., 1998; Köbel et al., 2008). Recently, CDH6 was found to be overexpressed in aggressive papillary thyroid cancer (PTC) both *in vitro* and in tissue derived from PTC patients (Ciarrocchi et al., 2011; Sancisi et al., 2013). The authors found that expression of CDH6 is localized to the invasive front of tumors and controlled by TGFβ induced RUNX2-regulated transcription of SNAI 1/2. Notably, CDH6 expression is specific to thyroid cancer cells as compared to wild-type thyrocytes. Follow-up work by Gugnoni and colleagues found that loss of CDH6 expression by siRNA attenuates the mesenchymal features of thyroid cancer cells by altering cytoskeletal architecture and intercellular associations (Gugnoni et al., 2017). Using a yeast-two hybrid screen and GST-pulldown experiments, they identified interactions between CDH6 and three autophagy related proteins, GABA type A receptor-associated protein (GABARAP), BCL2 interacting protein 3 (BNIP3), and BCL2 interacting protein 3 like (BNIP3L). Subsequent work demonstrated that CDH6 restrains autophagy and promotes cell motility through dynamin-related protein (DRP1) mediated mitochondrial reorganization. To further support a role for CDH6, the authors analyzed a large cohort of tissue specimens from patients with thyroid cancer, and found that through these interactions, CDH6 expression independently associated with distant metastases and decreased disease specific survival in PTC patients. In this study, inhibition of autophagy is thought to be a consequence of CDH6 binding to GABARAP with concomitant DRP-1 mediated mitochondrial fission, and subsequent cytoskeletal remodeling, cell migration, and EMT induction. The model suggests that induction of autophagy in PTC cells could negatively control and ultimately reverse EMT by promoting mitochondrial fusion and cytoskeletal stabilization. Baculoviral IAP Repeat Containing 7 (BIRC7) is an inhibitor of apoptosis (IAP) family gene whose expression is linked to tumor progression and metastasis in lung and prostate cancer (Chen et al., 2012; Zhuang et al., 2015). BIRC7 has recently been shown to be upregulated in several validated PTC cell lines, as well as fresh tumor tissue from PTC patients, as compared to wildtype thyrocytes or benign thyroid tissue (Liu et al., 2020). In the same study, markers of EMT were significantly downregulated with knockdown of BIRC7 whereas overexpression markedly enhanced PTC migration and invasion *in vitro*. Conversely, markers of autophagy were increased with knockdown of BIRC7 and decreased with overexpression suggesting that BIRC7 works to suppress autophagy in PTC cells. To further dissect the link between autophagy and BIRC7, the authors assessed the effect of concomitant BIRC7 knockdown with either BECN1 or ATG5 knockdown in PTC cells and found that both migration and invasion were markedly increased, suggesting that the induction of autophagy resulting from BIRC7 knockdown is protective in PTC. They then compared the effect of rapamycin, a well-described drug known to induce autophagy and found that BIRC7 knockdown together with rapamycin synergistically reduced cell migration and invasion while increasing autophagy flux. Markers of EMT were similarly significantly decreased with dual rapamycin therapy with BIRC7 knockdown. Importantly, *in vivo* xenograft models of PTC metastasis with tail vein injection showed a significantly reduced burden of metastatic disease in animals injected with BIRC7 knockdown PTC cells co-treated with rapamycin. Once again, markers of autophagy activation were upregulated and markers of EMT were downregulated in mice injected with BIRC7 knockdown PTC cells and treated with rapamycin. Taken together, these findings suggest that BIRC7 promotes invasion and metastasis by driving EMT and inhibiting autophagy.

Notably, a recent study evaluating the effect of autophagy inhibition in thyroid cancer found that vimentin, a marker of mesenchymal cells, was significantly downregulated in both WDTC and ATC cells treated with Lys05, a potent and specific inhibitor of lysosomal autophagy (Holm et al., 2021). Directed autophagy inhibition with Lys05, chloroquine, or FIP200 (a mediator of canonical autophagy) knockdown decreased cell migration and invasion thereby attenuating the effects of EMT rather than promoting EMT progression. These findings contrast with those of Liu and Gugnoni where they found that restraining autophagy in the context of CDH6 and BIRC7 upregulation was a possible driver of the mesenchymal phenotype.

The relationship between EMT and autophagy in the pathogenesis of cancer continues to be controversial and incompletely defined. Multiple studies have demonstrated that both autophagy inhibition and induction may modulate EMT in conflicting ways (Gugnoni et al., 2017; Liu et al., 2020; Holm et al., 2021; Hou et al., 2021). The stimulus driving either EMT or autophagy, together with the specific tumor microenvironment likely affects the ultimate outcome. Interestingly, in colorectal cancer cells, both induction of autophagy by mTOR inhibition or inhibition of autophagy by BECN1 knockdown lead to inhibition of EMT (Gulhati et al., 2011; Ren et al., 2015; Shen et al., 2018). Whether autophagy induction or inhibition is a consequence of EMT activation, or whether autophagy functions as a driver, or an inhibitor of EMT in thyroid cancer remains to be determined.

### Apoptosis and Autophagy: to Die or to Devour?

Programmed cell death type 1, or apoptosis, is induced by multiple stimuli including cellular stress, DNA damage, and immune surveillance (Das et al., 2021). Dysregulation of apoptosis has been well described in cancer biology. Defective apoptosis can drive *de novo* tumor development resulting from a failure of normal cell turnover and also promote tumor progression and metastasis by supporting: 1) a more permissive environment for genetic instability, 2) resistance to immune cell and cytotoxic (chemo/radiation) therapies, and 3) tumor cell survival despite a hypoxic or detached/suspended (metastatic) state.

The relationship between autophagy and cell death is complex because autophagy can drive both an adaptive, pro-survival response as well as a cytotoxic, pro-death response (Das et al., 2021). Whether a given cell will initiate apoptosis or autophagy depends upon the cellular context, intensity of the stimulus, and the cell’s threshold for either response. Cross-talk between autophagy and apoptosis is well-described (Das et al., 2021). As with EMT, both pathways are induced by many of the same stressors (reactive oxygen species, p53, death associated protein kinases, BH3-only proteins). Moreover, several canonical autophagy proteins including ATG5 and BECN1 directly and indirectly impact the regulation or execution of the apoptotic pathway (Das et al., 2021). Autophagy and apoptosis are for the most part mutually inhibitive but whereas apoptosis is purely a cell death pathway, autophagy can induce either cell survival or cell death.

Therapeutic strategies to induce apoptosis in cancer cells is of significant interest in cancer research. Both from the perspective that the apoptosis machinery is often mutated during tumorigenesis and because therapeutic resistance is frequently associated with ineffective apoptosis and/or necrosis of tumor cells. Suppression of apoptosis can induce autophagy, which in turn may promote tumor survival by supporting cell growth and proliferation in a nutrient-poor, hypoxic environment. Paradoxically, autophagy induction may also support tumor cell death by promoting tumor regression through type II programmed cell death (PCD) or type III cell death, necrosis. Multiple non-apoptotic programmed cell death pathways also exist which interact with autophagy, including necroptosis, ferroptosis, and pyroptosis (Miller et al., 2020). A deeper understanding of the relationship between cancer biology, autophagy, apoptosis, and necrosis is important for the development of novel, effective therapeutics.

Specific to thyroid cancer, the relationship between autophagy, apoptosis and cell necrosis is complex. There are a wealth of studies which demonstrate both: 1) a pro-tumorigenic effect of autophagy with increased cell proliferation and concomitant suppression of apoptosis and/or cell death and 2) a tumor suppressive effect of autophagy with decreased cell proliferation, induction of apoptosis and/or cell death (Li et al., 2014; Ren et al., 2015; Feng et al., 2018; Das et al., 2021). Regarding several autophagy modulators, findings with respect to upregulation vs. downregulation of autophagy as well as subsequent effects on apoptosis, cell proliferation, and cell death, often conflict across different thyroid cancer types and sometimes between different studies.

BRAF-activated long non-coding RNA (BANCR) was initially shown to be an overexpressed inducer of EMT in melanoma, colon cancer, and non-small cell lung cancer (Sunaga and Kaira, 2015; Yu et al., 2017; Hussen et al., 2021). Specific to thyroid cancer, BANCR was found to be uniquely expressed both in PTC cells *in vitro* and in tumor samples derived from PTC patients as compared to normal controls (Wang et al., 2014). Overexpression of BANCR in PTC cells markedly activated autophagy and inhibited apoptosis but there was no effect on cell migration or invasion. Knockdown of BANCR inhibited cell proliferation and increased apoptosis in PTC cells. Inhibition of autophagy stimulated apoptosis and abrogated the effects of BANCR on cell proliferation. These results suggest that autophagy stimulation induced by BANCR is tumor protective in thyroid cancer because induction of autophagy with BANCR led to increased cell proliferation and decreased apoptosis.

Another protein known to modulate autophagy and apoptosis in thyroid cancer is Bcl2-associated athanogene 3 (BAG3) (Li et al., 2014). BAG3 was initially identified as an anti-apoptotic protein and negative regulator of EMT expressed in multiple thyroid cancer cell lines (Chiappetta et al., 2007; Meng et al., 2014). Subsequent studies demonstrated a role for BAG3 in the activation of chaperone-mediated autophagy in HepG2 cells derived from a patient with hepatocellular carcinoma (Liu et al., 2013). In an elegant follow-up study aiming to understand whether BAG3 might also be involved in canonical autophagy in thyroid cancer cells, Li et al. (2014) sought to evaluate the effects of BAG3 expression under starvation conditions thought to mimic the tumor microenvironment. Under starvation conditions, they found that autophagy was induced and BAG3 expression inhibited. However, the forced expression of BAG3 under these same conditions suppressed autophagy and promoted apoptosis (Liu et al., 2013; Li et al., 2014). The duality of BAG3, promoting cell survival *via* upregulation of CMA (Liu et al., 2013) while also promoting apoptosis via inhibition of canonical autophagy, (Li et al., 2014) is likely reflective of the cellular context, the underlying autophagy stimulus, and the complex nature of autophagy modulation itself.

An additional study highlighting the contradictory nature of autophagy modulation in thyroid cancer progression was recently published by Hou et al. (2021). They identified lactate dehydrogenase A (LDHA), an enzyme overexpressed in aggressive PTC, as a driver of EMT gene transcription, cell proliferation and autophagy regulator (Hou et al., 2021). LDHA was identified as a potential diagnostic and therapeutic target for PTC following examination of the TCGA database. Overexpression of LDHA was confirmed in PTC tissues derived from patients with aggressive disease as well as multiple, well-established thyroid cancer cell lines. The authors found that LDHA promoted EMT, as well as cell proliferation *in vitro* and *in vivo*, in part by inhibiting autophagy. They observed that LDHA overexpression led to the inhibition of AMPK-mediated protective autophagy and apoptosis, with concomitant increased tumor cell proliferation. Knockdown of LDHA or the LDHA inhibitor, FX11, induced autophagy and inhibited apoptosis. Dual therapy with the LDHA inhibitor, FX11, and the autophagy inhibitor, hydroxychloroquine (HCQ), had a synergistic effect on apoptosis *in vitro* and significantly reduced tumor growth in mouse models.

This study is one of many which highlight the Janus-faced nature of autophagy. While LDHA overexpression induced a pro-tumorigenic phenotype with autophagy inhibition, upregulation of EMT and cell proliferation, induction of autophagy from LDHA knockdown or FX11 also promotes a pro-tumorigenic phenotype by inhibiting apoptosis in tumor cells. Therapy induced autophagy is well described (Lin et al., 2009; Ma et al., 2011; Zhang et al., 2015) with several studies highlighting the effect of “enforced” autophagy resulting from therapeutic influence. The role of autophagy modulation and apoptosis in the context of therapeutic interventions for thyroid cancer is an active area of investigation.

Vemurafenib is a selective RAF inhibitor utilized in patients with BRAF-mutant melanoma (Wang et al., 2017). However, while the BRAFV600E mutation is common in aggressive forms of thyroid cancer, there is significant resistance to vemurafenib therapy in this patient population. An elegant study by Wang et al. (2017) found that this observation may result from the induction of autophagy by vemurafenib. They found that dual therapy with vemurafenib and the autophagy inhibitor HCQ augmented vemurafenib-induced cell death by blocking ER stress-response mediated autophagy both in cell culture and xenograft mouse models.

Similar work evaluating another well described tyrosine kinase inhibitor, apatinib, found that induction of autophagy by apatinib led to inhibition of apoptosis and therapeutic resistance (Feng et al., 2018; Meng et al., 2020). Dual therapy with apatinib and autophagy inhibition with either HCQ or CQ led to reduced tumor growth and augmented apoptosis both *in vitro* and *in vivo* in PTC and ATC models (Feng et al., 2018; Meng et al., 2020). In another study evaluating the effect of the multi-kinase inhibitor, sorafenib, in thyroid cancer found that dual therapy with the autophagy inhibitor CQ significantly suppressed tumor growth as compared to either therapy alone (Yi et al., 2018). Moreover, the authors demonstrated that while sorafenib alone increased both autophagic activity and apoptosis, the introduction of an autophagy inhibitor served to specifically augment apoptosis in both ATC and FTC cell lines and in xenograft models.

Notably, these studies contrast with older work which suggested that dual therapy with autophagy enhancement, as opposed to inhibition, and antitumor therapy served to augment tumor cell death in thyroid cancer. Lin et al. (2009) found that while autophagy was significantly induced by radiation or doxorubicin, inhibition of autophagy actually decreased both radio- and chemo-sensitivity. In follow up to this, they found that enhanced autophagy with RAD001, a potent activator of autophagy, sensitized PTC cells to cytotoxic chemotherapy with doxorubicin and radiation (Lin et al., 2010). Importantly, the authors found that the increased cytotoxicity stemming from dual therapy with RAD001 and either doxorubicin or radiation did not correlate with a concomitant increase in apoptosis but rather an attenuation of c-MET signaling. Whether the synergistic affect of autophagy induction with doxorubicin or radiation drives increased autophagic cell death, necrosis, or other non-apoptotic cell death remains to be determined.

A more recent study looking at the effects of autophagy inhibition alone found that there was no effect of autophagy inhibition on apoptosis in two PTC cell lines (Holm et al., 2021). However, in FTC and ATC cell lines, inhibition of autophagy with Lys05 was associated with induced apoptosis *in vitro* (Holm et al., 2021). These differences in the effects of autophagy modulation likely highlight the inherent distinctions in the pathogenesis of different thyroid cancer types as well as differences in native to the specific autophagy stimulus.

### Cancer Stem Cells and Autophagy: Maintaining the Quietest Cell

Within solid tumors, cancer stem cells (CSCs) exist as a small subpopulation of cancer cells that retain the capacity for long-term self-renewal, proliferation, and multi-potent differentiation (Hardin et al., 2017; Nazio et al., 2019). The CSC hypothesis posits that CSCs are responsible for tumor heterogeneity, proliferation, invasion, and metastasis. In this model, tumorigenesis, recurrence, and therapeutic resistance are the result of a small but persistent CSC population which fails to respond to conventional anticancer regimens. Targeted therapy with complete elimination of CSCs would therefore address both continued tumor growth and relapse.

Autophagy has been proposed to play a role in the maintenance of CSC pluripotency as well as the CSC niche. Failure of autophagy in multiple cancer types: breast, pancreas, liver, ovarian, glioblastoma leads to the negative regulation of staminal markers of CSCs which ultimately influence their self-renewal ability (Gong et al., 2013; Song et al., 2013; Wolf et al., 2013; Yeo et al., 2016; Zhang et al., 2016; Nazio et al., 2019). Elegant work by Gong et al. (2013) identified a role for autophagy in the maintenance of CSCs in breast cancer. They found that autophagy was upregulated in breast CSCs as compared to bulk tumor. Inhibition of autophagy mediated by Beclin-1 shRNA significantly reduced breast CSC proliferation and self-renewal. Xenograft experiments with breast CSCs with or without shRNA induced silencing of beclin-1 demonstrated that autophagy is critical for tumorigenicity. Subsequent work in pancreatic and ovarian cancer have similarly identified autophagy as an essential player in the maintenance and tumorigenicity of CSCs (Yang et al., 2015; Buccarelli et al., 2018). Altogether, these studies found that combined therapy with autophagy inhibition and standard chemotherapy had a synergistic effect, eradicating or significantly reducing tumor development. As such, inhibition of autophagy, with its concomitant destabilization of the CSC population, provides an attractive therapeutic target.

Thyroid CSCs (TCSCs) are characterized by the expression of various biomarkers including ALDH and CD44 as well as their ability to form thyrospheres *in vitro* and tumors *in vivo* (Todaro et al., 2010). They are highly tumorigenic and recapitulate the behavior of the parental tumor with respect to their ability to locally invade and metastasize. Across different thyroid cancer subtypes, CSCs are most common in ATC (∼5%), followed by PTC (∼2%) and FTC (1%–2%). As with most CSCs, TCSCs are quiescent and intrinsically resistant to conventional therapy. These features of TCSCs are thought to drive drug resistance, metastasis, and recurrence. Targeted therapeutics aimed to eliminate TCSCs could either directly eradicate the CSCs themselves or induce or promote terminal differentiation.

The underlying mechanisms which specifically support TCSC maintenance are an area of active investigation. A great deal of information has been inferred by observing how both the tumor microenvironment and pathways crucial to TCSC viability are modified. Within the tumor microenvironment, the TCSC niche is sustained by a complex, bidirectional network of chemokines/cytokines mediated by the surrounding stroma including cancer associated fibroblasts (CAFs), macrophages, and lymphocytes (Visciano et al., 2015; Parascandolo et al., 2017; Zheng et al., 2019). Dysregulation of the NF-kB, MAPK, and GSK3b pathways have been implicated in the maintenance of TCSCs themselves across multiple thyroid cancer subtypes (Ricarte-Filho et al., 2009; Li et al., 2013). Because EMT is a clear example of cellular plasticity, which is also a feature of CSCs, multiple studies have also sought to investigate the relationship between EMT and TCSCs. Interestingly, their work has revealed a correlation between EMT upregulation and increased TCSCs in PTC, FTC, and ATC (Liu and Brown, 2010; Lan et al., 2013).

Epigenetic modifications thought to maintain TCSC viability include: 1) DNA methylation 2) histone modification 3) non-coding RNA including miRNAs and lncRNAS (Vu-Phan and Koenig, 2014). DNA methylation has been implicated as a principal pathway because of the observation that components of the principal pathways involved in TCSC survival, MAPK, PI3K, and beta-catenin, are silenced by hypermethylation (Vu-Phan and Koenig, 2014). While the methylome profile of TCSCs is not yet defined, a role for DNA methylation in TCSC viability is hypothesized because of the focus on methylation of the promoters of thyroid-specific differentiation (Borbone et al., 2011). Histone deacetylase (HDAC) inhibitors are a well-known treatment for impairing cell growth, inducing apoptosis, and increasing radioiodine uptake in thyroid cancer (Russo et al., 2013). Bromodomain containing (Singh et al., 2018) (BRD4), lysine demethylase 1a (KDM1A), and enhancer of zeste homolog 2 (EZH2) are histone modifiers which have all been recently implicated as drivers critical for TC proliferation, invasion, and migration (Borbone et al., 2011; Gao et al., 2016; Zhang et al., 2019). Non-coding RNAs have long been recognized as critical regulators of EMT and CSCs in general. Several miRNAs and lncRNAs have found to be dysregulated in multiple subtypes of TC. Recently, two lncRNAs, lncRNA -LINC00311 and lncRNA-H19 were identified as specific promotors of cancer stem-like properties in PTC (Li et al., 2018; Gao et al., 2020). Similarly, two miRNAs: miR-148a and antisense-miR-21, were both found to significantly inhibit TCSCs in ATC cells (Haghpanah et al., 2016; Sheng et al., 2016).

Data regarding the specific relationship between autophagy and thyroid CSCs is limited and largely inferred from experiments seeking to investigate mediators of thyroid CSC viability. For example, high-mobility group box 1 (HMGB1), a highly conserved DNA-binding protein known to influence autophagy, CSC survival, and EMT was recently found to play a role in thyroid cancer (Kang et al., 2013; Chai et al., 2019). HMGB1 was identified as a critical regulator of autophagy-mediated NIS degradation in differentiated thyroid cancer. Knockdown of HMGB1 suppressed autophagy and sodium iodide symporter (NIS) degradation both *in vitro* and in xenograft mouse models (Chai et al., 2019). As NIS expression is a marker of differentiation in thyroid cells, it is inferred that HMGB1 has a role in TCSC survival. One recent study evaluated the direct effect of autophagy inhibition on CSC maintenance (Holm et al., 2021). They found that direct inhibition of autophagy with Lys05 or chloroquine led to almost complete abrogation of thyrosphere formation in PTC, FTC, and ATC cells *in vitro*. Further, knockdown of FIP200 significantly reduced ALDH activity and CD44 expression. Interestingly, autophagy inhibition with Lys05 mitigated cell migration, invasion, and proliferation. Vimentin expression, a marker of EMT, was also reduced. Taken together, these data would suggest that TCSCs are indeed drivers of a more aggressive phenotype (upregulated EMT, migration, invasion) which is, at least in part, supported by autophagy.

## Conclusion and Future Directions

Thyroid cancer continues to be a major public health issue with almost one million people living with the disease in the United States alone. Autophagy clearly plays a role in the pathogenesis of disease and remarkable progress has been made regarding the relationship between modulation of autophagy and progression of thyroid cancer (Table 1). Emerging evidence supports a possible role for autophagy inhibition either alone or in concert with targeted therapies (e.g., sorafenib, apatinib) as a means to induce cell death and/or apoptosis while also inhibiting proliferation, invasion, and migration (Feng et al., 2018; Yi et al., 2018; Meng et al., 2020). Clarifying the role of autophagy in TCSC viability and maintenance is particularly attractive as their prolonged quiescent state and resistance to conventional therapies likely contributes to both recurrence and persistence in thyroid cancer. Seemingly contradictory observations regarding the implications of autophagy modulation underscore our incomplete understanding of the complex cross-talk between the various molecular pathways involved and how cellular context regarding timing and autophagy stimulus (starvation vs. chemical) affect outcomes. Future studies which elucidate the molecular mechanisms underlying the crosstalk between autophagy, EMT, apoptosis, and TCSC viability will allow for the development of targeted, selective and individualized treatment strategies to eradicate thyroid cancer.
